# Prevalence and associated factors of mental disorders in the nationwide primary care population in Latvia: a cross-sectional study

**DOI:** 10.1186/s12991-020-00276-5

**Published:** 2020-04-07

**Authors:** Elmars Rancans, Lubova Renemane, Anda Kivite-Urtane, Douglas Ziedonis

**Affiliations:** 1 Department of Psychiatry and Narcology, Riga Stradins University, 2 Tvaika Str, Riga, LV-1005 Latvia; 2 Department of Public Health and Epidemiology, Riga Stradins University, 9 Kronvalda Ave, Riga, LV-1010 Latvia; 3 University of California San Diego, Biomedical Sciences Building, Room 1310, 9500 Gilman Drive #0602, La Jolla, San Diego, CA 92093 USA

**Keywords:** Depression, Anxiety, Primary care, Mental disorders, Prevalence

## Abstract

**Background:**

Mental disorders are common amongst patients in primary care. There are no published studies on the prevalence of mental disorders in primary care patients in Latvia. The purpose of the study was to evaluate the current prevalence of mental disorders in the nationwide Latvian primary care population and to study possible associated factors and comorbidity of mental disorders.

**Methods:**

A cross-sectional study within the framework of the National Research Program BIOMEDICINE 2014–2017 was performed at 24 primary care settings across Latvia. Adult patients seen over a 1-week time period at each facility were invited to participate in the study. Sociodemographic variables (age, sex, education, employment and marital status, place of residence, and ethnicity) were assessed onsite. A Mini-International Neuropsychiatric Interview assessment was conducted over the telephone within 2 weeks after the visit to the general practitioner (GP).

**Results:**

Overall, 1485 individuals completed the interview. The current prevalence of any mental disorder was 37.2% and was significantly greater in women. Mood disorders (18.4%), suicidality (18.6%) and anxiety disorders (15.8%) were the most frequent diagnostic categories. The current prevalence of any mood disorder was associated with being 50–64 years of age, female sex, economically inactive status, divorced or widowed marital status and urban place of residence, whilst any current anxiety disorder was associated with female sex, lower education, and single marital status; however, being of Russian ethnicity and residing in a small city were protective factors. Suicidality was associated with female sex, lower education, unemployment or economically inactive status, being divorced or widowed and residing in a small city. The comorbidity rates between mental disorders varied from 2.9 to 53.3%.

**Conclusions:**

High prevalence rates of mental disorders, comorbidity and certain associated socio-demographic factors were found in primary care settings in Latvia. This highlights the importance of screening for depression and anxiety disorders and suicidal risk assessment by GPs. The results are fundamentally important for integrative medicine, monitoring and promotion of mental healthcare at the primary care level, as well as for healthcare policy and development of strategic plans in Latvia.

## Background

The high prevalence of mental disorders worldwide is not only an important medical factor but also entails a number of negative social and economic burdens on society and potential impacts on quality of life, productivity, health-related work losses and increased healthcare costs [1–4]. People with mental disorders experience disproportionately higher rates of disability and mortality. The World Health Organization (WHO) report of mental health indicates that persons with major depression and schizophrenia have a 40–60% greater chance of dying prematurely than the general population, owing to physical health problems that are often left unattended and suicide [5].

The prevalence of mental disorders in primary care settings in Europe has been estimated in various studies to be between 20 and 55% [6–10].

The general practitioner (GP) plays an influential role in the early diagnosis and treatment of common mental disorders, such as depression, anxiety, substance abuse and dependence in the primary care setting [6, 7]. The results of a large-scale population-based cohort study of primary care patients emphasized that systematic mental health screening followed by feedback to the GP regarding screening results can contribute to initiation and cessation of mental healthcare and raise awareness of the current needs of the patients [11].

However, the number of specialized and general health workers dealing with mental health in low-income and middle-income countries is grossly insufficient [11]. On the other hand, mental disorders are undertreated and underdiagnosed in primary care settings [12].

A comprehensive meta-analysis study consisting of 1 million participants from communities globally (30 countries from all continents) indicated a point prevalence of depression of 12.9%, a 1-year prevalence of 7.2% and a lifetime prevalence of 10.8% [13]. Another study of the general adult population in 21 countries reported a 12-month prevalence rate of depression ranging from 2.4 to 10.1% and suggested that only a minority of depressed patients receive treatment [14]. However, specific educational programmes for family physicians [15–17], providing information on Internet [18] and electronic health [19] can improve diagnostics of depression in primary care settings and decrease the prevalence of mental disorders over the long term [16].

The WHO World Mental Health (WMH) community surveys in 28 countries throughout the world documented a lifetime prevalence of anxiety disorders ranging between 5 and 25% of the population and a 12-month prevalence ranging between 3.3 and 20.4% [3]. The 12-month prevalence of anxiety disorders was 9.8% in recent WHO WMH community surveys in 21 countries. In the same study, it was found that only 27.6% of these patients received any treatment, and only 9.8% received potentially adequate treatment. In addition, only 41.3% of those in the 12-month prevalence group perceived a need for care [12].

Previous studies have explored the comorbidity of common mental disorders in the primary care setting and emphasize that depressive disorders are highly associated with anxiety disorders [6, 20]. In another study, it was shown that one-half of outpatients identified by their family physician as having a depressive disorder also have a comorbid mental disorder, usually an anxiety disorder (48.6%), with social phobia being the most common (25.3%) [21].

It is important to state that the presence of a mental disorder, such as mood disorders, anxiety disorders, substance use disorders and schizophrenia, is one of the risk factors for suicidal ideation [22–24]. Suicidal ideation is highly prevalent in the general population (8.5%) [25] and in primary care samples (18–32%) [26], and has been identified as a predictor for death by suicide [27, 28]. These facts underline the necessity of early detection and evaluation of risk factors of common mental disorders, especially in the primary care population [29].

Reliable epidemiological data on the prevalence of common mental disorders in Latvia are limited and often based on expert estimates and opinions. The first ever-population-based study in Latvia reported a point prevalence of depression of 6.7% [30]. A few years later, the 12-month prevalence of depression was estimated to be 7.9% in a population-based study in Latvia that reported the following factors associated with depression: frequent use of healthcare services, somatic comorbidity, dissatisfaction with health status, and occasional smoker status [31].

One of the four major objectives of the WHO Mental Health Action Plan 2013–2020 is to provide comprehensive, integrated and responsive mental health and social care services in community-based settings [5].

Whilst the primary care sector presently provides management, diagnosis and treatment of a large cluster of common mental disorders, there are currently no Latvian studies investigating the prevalence of mental disorders and associated factors in the primary care population.

Therefore, the first aim of this study was to report the current prevalence of mental disorders and suicidality in the nationwide Latvian primary care population, identify sociodemographic characteristics that may be associated with mental disorders in primary care settings and explore the comorbidities of mental disorders in this sample.

In addition, the results of this study are fundamentally crucial to integrative medicine and the promotion of mental healthcare at the primary care level, as well as for healthcare policy, medical education and the development of programmes in Latvia and the Baltic states.

## Methods

### Study design

We performed a cross-sectional study in 2015 within the framework of the National Research Program BIOMEDICINE 2014–2017 to assess the prevalence of common mental disorders in the Latvian primary care population. The sampling frame was all health regions of Latvia, and respondents were recruited from 24 primary care facilities (16 in urban and 8 in rural areas). All consecutive patients with an appointment with any of the participating family physicians during a 1-week period at each primary care facility were invited to participate in the study.

Patients were eligible if they were treatment-seeking patients visiting a GP, were aged 18 years or older, and had provided their informed consent. We excluded patients who refused to participate in the study, who were unable to participate due to their somatic condition (e.g., being deaf-mute), who had an acute medical condition requiring urgent hospitalization or who were visiting their GP for administrative reasons.

Before seeing their GP and after signing the informed consent, all consecutive participants were asked to complete a sociodemographic questionnaire during a 1-week period at each primary care facility. Four trained psychiatrist interviewers conducted the Mini-International Neuropsychiatric Interview (MINI), Version 6.0.0, over the phone within a period of 2 weeks after the first contact with the patient.

### Assessment tools

The MINI is a standardized and short-structured diagnostic interview for epidemiology studies and is used for evaluation of mental disorders according to the DSM-IV and International Classification of Disease 10th version (ICD-10) in psychiatric populations and in general medical populations including primary care patients [32, 33]. The validation of the MINI was performed in relation to the Structured Clinical Interview for DSM-III-R, Patient Version, the Composite International Diagnostic Interview, and expert professional opinion [32]. The MINI has been translated and adapted by the authorship holders for use in 67 languages, including Latvian and Russian [34]. Administration time of the MINI was approximately 15 min and the interview was conducted over the telephone, which is acceptable and has been carried out by other studies [35, 36]. We used the MINI modules to identify current diagnoses of major depressive episode, recurrent depressive disorder, mania, hypomania, bipolar disorder I, bipolar disorder II, suicidality, psychotic disorder, posttraumatic stress disorder, panic disorder, social phobia, generalized anxiety disorder, agoraphobia, obsessive–compulsive disorder, alcohol dependence, alcohol abuse, anorexia, and bulimia.

The sociodemographic questionnaire included questions about sex, age group (18–34 years, 35–49 years, 50–64 years, and 65+ years), marital status (married, not married), educational level, ethnicity and employment. For educational level, the participants were categorized into three groups: (1) higher or unfinished higher education, (2) general or vocational secondary or unfinished secondary education, and (3) 9-year basic, unfinished basic education.

### The characteristics of participants

We invited a total of 1756 patients who visited their GP to participate in the study, and 152 refused to participate. The mean study response rate was 91.3% and varied from 86.3 to 93.7% across 24 primary care settings throughout Latvia. Those who refused to participate did not differ significantly in basic sociodemographic characteristics from the study sample.

At baseline, a sample of 1604 patients was approached to complete the questionnaire. The questionnaire was fully completed by 1585 participants. Of those who completed the questionnaire, 100 patients did not answer a follow-up telephone call three times and were excluded from the study. A total of 1485 patients were interviewed with the MINI over the telephone.

The sociodemographic characteristics of the subjects are shown in Table 1. The study participants were predominantly female (69.5%), had general or vocational secondary or unfinished secondary education (57.4%), were employed (53.2%), were married (61.4%), lived in a small city (47.3%) and were of Latvian ethnicity (62.3%).

**Table 1:** Sociodemographic characteristics of study sample (*n = *1485)

Sociodemographic characteristics	N	%
Age		
18–34	211	14.2
35–49	462	31.1
50–64	354	23.8
65+	458	30.8
Sex		
Male	453	30.5
Female	1032	69.5
Education^a^		
Higher and unfinished higher education	442	29.9
General or vocational secondary and unfinished secondary	848	57.4
9-year basic, unfinished basic	187	12.7
Employment status^a^		
Employed	787	53.2
Unemployed	84	5.7
Economically inactive	607	41.1
Marital status^a^		
Married, cohabiting	907	61.4
Single	144	9.7
Live separately, divorced, widowed	427	28.9
Place of residence		
Capital (Riga)	309	20.8
Other city	702	47.3
Rural	474	31.9
Ethnicity^a^		
Latvian	920	62.3
Russian	463	31.3
Other	94	6.4

^a^The sum of stratified numbers may differ across variables due to missing values

### Statistical analysis

Data were analysed using Statistical Package for the Social Sciences (SPSS) version 20.0 (IBM SPSS Corp.). Descriptive statistics, including means, standard deviations, frequencies and 95% confidence intervals, were used to describe the data. Statistically significant differences in the distribution of variables between sexes were detected using a Chi square test or Fisher’s exact test. Factors associated with mental disorders were identified using binary logistic regression. Statistical significance was defined as *p* < 0.05.

## Results

### Prevalence of current mental disorders

Additional file 1: Table S1 shows the current prevalence of mental disorders established by the MINI. The current prevalence of any mental disorder was 37.2% (95% CI 34.7–39.7) and was significantly higher in women (p < 0.001). Any mood disorder (18.4%; 95% CI 16.4–20.4), suicidality (18.6%; 95% CI 16.6–20.6) and any anxiety disorder (15.8%; 95% CI 13.9–17.7) were the most frequent diagnostic categories.

Recurrent depressive disorder 17.5% (95% CI 15.6–19.4), suicidality with low risk 17.1% (15.2–19.0), depressive episode 10.2% (95% CI 8.7–11.7), agoraphobia 8.0% (95% CI 6.6–9.4) and generalized anxiety disorder 6.1% (95% CI 4.9–7.3) were the most common psychiatric disorders in our sample.

During the last 30 days 1.9% (95% CI 1.3–2.7) of respondents experienced suicidal ideas, 0.5% (95% CI 0.2–1.0) had a suicidal plan and 0.1% (95% CI 0.01–0.4) reported the history of suicidal attempts with no statistically significant differences between sexes.

The results demonstrated several clear sex differences. Recurrent depressive disorder, suicidality, depressive episode, agoraphobia and generalized anxiety disorder were significantly more frequent amongst females than males. However, alcohol dependence and abuse were significantly more frequent in males.

The majority of mood disorders consisted of depressive episode and recurrent depressive disorder. Mania was not reported in our sample. Only bulimia patients reported any eating disorder in the sample.

Although the main aim of this study was current prevalence of mental disorders, according to the MINI several lifetime prevalence could be also assessed. Criteria for lifetime depression were met by 28.1% (95% CI 25.9–30.4) of respondents (females vs. males, 32.4% and 18.3% accordingly, *p < *0.001), and lifetime recurrent depressive disorder by 17.5% (95% CI 15.6–19.5) (females vs. males, 19.9% and 11.9% accordingly, *p* < 0.001). Lifetime history of any psychotic disorder was reported by 3.8% (95% CI 2.9–4.9) of respondents and 4.1% (95% CI 3.2–5.3) for lifetime suicidal attempt with no statistically significant differences between sexes. 3.4% (95% CI 2.6–4.5) of respondents met the criteria for lifetime panic attack (males vs. females 1.8% and 4.2%, *p* = 0.02). Whereas 0.5% (95% CI 0.2–1.0) of the respondents were diagnosed as having a lifetime Bipolar I and 0.9% (95% CI 0.6–1.6)—Bipolar II disorders with no statistically significant differences between sexes.

### Factors associated with current mental disorders

Additional file 2: Table S2 presents the sociodemographic factors associated with current mental disorders. In the adjusted analyses (adjustment performed for age and sex), the factors statistically associated with any mental disorders were female sex (OR = 1.61, 95% CI 1.27–2.04), low education (vs. higher education; OR = 1.83, 95% CI 1.26–2.65), unemployed (vs. employed; OR = 1.65, 95% CI 1.04–3.10), economically inactive employment status (vs. employed; OR = 1.47, 95% CI 1.09–2.00), marital status of being single (vs. married; OR = 1.59, 95% CI 1.08–2.36) and living separately, being divorced or widowed (vs. married; OR = 1.38, 95% CI 1.08–1.77).

Higher odds of any mood disorder were linked to factors of being between 50- and 64-year old (vs. 18- to 34-year old; OR = 1.64, 95% CI 1.04–2.58), female sex (OR = 1.92, 95% CI 1.40–2.65), economically inactive status (vs. employed; OR = 1.51, 95% CI 1.04–2.18), marital status of living separately, divorced or widowed (vs. married; OR = 1.77, 95% CI 1.31–2.39) and urban place of residence (capital of Latvia vs. rural; OR = 1.89, 95% CI 1.28–2.79; other city vs. rural; OR = 1.86, 95% CI 1.34–2.59).

Higher odds of current suicidality were associated with female sex (OR = 1.66, 95% CI 1.22–2.25), lower education (vs. higher education; OR = 2.38, 95% CI 1.55–3.67), unemployed (vs. employed; OR = 1.78, 95% CI 1.03–3.10), economically inactive employment status (vs. employed; OR = 2.39, 95% CI 1.66–3.45), marital status of being single (vs. married; OR = 2.13, 95% CI 1.35–3.34), living separately, being divorced or widowed (vs. married; OR = 1.50, 95% CI 1.10–2.03) and residence in a small city (vs. rural; OR = 1.52, 95% CI 1.11–2.09).

Higher odds of current any anxiety disorder were found for females (OR = 1.67, 95% CI 1.20–2.32), persons with lower education (general or vocational secondary or unfinished secondary, 9-year basic or unfinished basic vs. higher education, OR = 1.84, 95% CI 1.292.63), and single marital status (vs. married; OR = 2.36, 95% CI 1.433.90). We found that being of Russian origin (vs. Latvian; OR = 0.69, 95% CI 0.500.96) and residing in a small city (vs. rural; OR = 0.67, 95% CI 0.500.97) were protective factors for any anxiety disorder.

The sole factor statistically associated with any psychotic disorder was economically inactive employment status (vs. employed; OR = 3.52, 95% CI 1.15–10.79).

Comparing to the age group 18–34, the age groups above 35 years have decreasing association with any alcohol use disorder. Factors significantly associated with alcohol dependence and abuse were residence in the capital city (vs. rural; OR = 4.06, 95% CI 1.998.28), marital status of being single (vs. married; OR = 2.81, 95% CI 1.445.49) and living separately, being divorced or widowed (vs. married; OR = 1.98, CI 1.023.84). Female sex (OR = 0.16, 95% CI 0.090.27) was a protective factor for alcohol abuse and dependence in our study. Being a member of other ethnic groups, excluding Russians (vs. Latvian; OR = 3.93, 95% CI 1.0115.34), increased the risk of having an eating disorder.

### Comorbidity amongst current mental disorders

It is important to note the high level of comorbidity between mental disorders. The proportion of patients having any mood disorder, suicidality, any anxiety disorder, any psychotic disorder, any alcohol disorder or any eating disorder who also met criteria for a diagnosis in another diagnostic group are shown in Table 2.

**Table 2:** Prevalence (%) of comorbidity between diagnostic groups

Baseline diagnosis	Prevalence (95%CI)					
	Any mood disorder	Suicidality	Any anxiety disorder	Any psychotic disorder	Any alcohol disorder	Any eating disorder
Any mood disorder *n* = 272		50.0 (44.1–55.9)	37.9 (32.1–43.7)	2.2 (0.5–3.9)	8.5 (5.2–11.8)	2.9 (0.9–4.9)
Suicidality *n* = 276	50.0 (44.1–55.9)		33.3 (27.7–38.9)	4.4 (2.0–6.8)	9.1 (5.7–12.5)	1.8 (0.2–3.4)
Any anxiety disorder *n* = 235	44.2 (37.9–50.6)	39.1 (32.9–45.3)		2.6 (0.6–4.6)	8.5 (4.9–12.1)	0.4 (0–1.2)
Any psychotic disorder *n* = 24	25.0 (7.7–42.3)	50.0 (30.0–70.0)	25.0 (7.7–42.3)		16.7 (1.8–31.6)	0
Any alcohol use disorder *n* = 70	32.9 (21.9–43.9)	35.7 (24.5–46.9)	28.6 (18.0–39.2)	5.7 (0.3–11.1)		2.9 (0–6.8)
Any eating disorder *n* = 15	53.3 (28.1–78.6)	35.7 (11.5–60.0)	6.7 (0–19.4)	0	13.3 (0–30.5)	

The most frequently co-occurring mental disorders were any mood disorder, suicidality and any anxiety disorder. Suicidality was the most frequent concurrent phenomenon in any psychotic disorder group. Any eating disorders were highly associated with any mood disorder

## Discussion

### Prevalence of mental disorders

To date, there have not been any studies investigating the prevalence of psychiatric disorders in primary care settings in Latvia. The present study highlights the relatively high current prevalence (37.2%, 95% CI 34.739.7) of any mental disorder amongst the primary care population. This result is rather consistent with other studies carried out in other countries [6, 7, 37–39]. However, the prevalence of any mental disorder in the Latvian primary care population appears even more elevated than in Lithuania (12-month prevalence 27%) [33]. The prevalence of mental disorders reported in studies conducted in primary care populations indicate that 46.3% of individuals suffered from a current mental disorder in Israel [39] and 42.5% in Belgium [7], with 31.2% experiencing a mental disorder in the past 12 months in Spain and 45.1% diagnosed with a lifetime mental disorder [6]. A study in the Arab world demonstrated a 42.3% rate of prevalence of mental conditions in primary care settings [40].

Our study confirmed previous findings that any mood disorders, especially depression, suicidality, and any anxiety disorders, are the most prevalent common psychiatric disorders in primary care [7, 33, 37, 40, 41].

The current prevalence of any mood disorder in our study, including all ICD-10 diagnoses, was 18.4%. The majority of cases of any mood disorder consisted of depressive episode and recurrent depressive disorder with current prevalence rates of 10.2% and 17.5% and lifetime prevalence rates 28.1% and 17.5%, respectively. This is consistent with other European [37, 39] and non-European studies [38]. The current and lifetime prevalence of depressive episodes in a Belgium primary care study based on the MINI were 10.3% and 28.1%, respectively [7]. Our findings are congruent with a study from Israel, in which 19.9% of primary care attenders had a current depressive disorder [39]. The prevalence rate we observed for depressive disorders is also comparable with the results obtained in a recent meta-analysis of communities from 30 countries. The results indicated that the aggregate point prevalence of depression was 12.9%, the 1-year prevalence was 7.2%, and the lifetime prevalence was 10.8% from 1994 to 2014 [13].

The current prevalence of any anxiety disorder, including panic disorders, agoraphobia, social phobia, obsessive–compulsive disorder, posttraumatic stress disorder, and generalized anxiety disorder was 15.8%. This prevalence is lower in our study than in a study conducted in Madrid (22.4%) [37] and in a Spanish primary care population-based study (12-month prevalence 18.49%) [6]. The results seem comparable to those of primary care attendees in Israel, in which the current prevalence of mental disorders and reported rates of any anxiety disorders was 19.4% [39]. Similar results were shown in a primary care-based Belgian study, where the prevalence rate was 16.1% [7].

The results of our study showed a rather high prevalence of suicidality (18.6%). Although a substantial proportion of cases were classified by the MINI as having a low risk of suicidality (17.1%), a more detailed analysis revealed that 10.7% of patients had at least death wishes, thoughts about suicide and some types of serious suicidal behaviours within the last month. In addition, every twelfth primary care patient (7.9%) presented with current feelings of hopelessness. Considering that a substantial proportion of suicide victims who die by suicide have been in contact with a primary care provider during the previous 30 days, timely recognition of such patients is very important to provide appropriate care [42]. Bunevicius et al. observed a 6% suicidal ideation rate in a cross-sectional survey-based study of 998 primary care patients that was three times lower than that seen in our study. However, the study was performed only in four primary care settings in two cities [33]. In contrast, Teismann et al. reported a 25% prevalence of suicidality in primary care patients suffering from panic disorder with or without agoraphobia [23].

The current prevalence of any psychotic disorder was 1.6% and lifetime prevalence was 3.8%. This result is consistent with other studies carried out in other countries in the primary care population [37, 38].

The current prevalence of alcohol dependence and abuse was 4.7%. These results seem comparable to those observed in a study conducted in the Madrid primary care population, with a prevalence rate of 4.4%. [37]. Although a primary care population study from Belgium found a higher prevalence of alcohol abuse and dependence, at 10.1%, the lifetime prevalence of alcohol abuse and dependence in community residents in Japan in 2010 was also higher, at 15.1% [43].

In a cross-sectional multi-centre study in six European countries, 358 general practitioners assessed 8476 patients in primary health care settings using multiple methods, resulting in an 8.7% prevalence of alcohol dependence. However, using the Composite International Diagnostic Interview (CIDI), the prevalence result was 5.5% in the same study [44].

However, differences in the results may be attributable partly to a differential sampling framework used in the studies and to the different sensitivity and specificity of diagnostic instruments (MULTICAGE CAD-4 questionnaire vs MINI vs PRIME-MD) and to the use of two measures versus one [45, 46]. Primary care screening of alcohol consumption could help primary and secondary prevention of alcohol use disorders. Most of the people with such disorders lack awareness of the problem, which prevents them from seeking treatment [44].

Regarding eating disorders, we found a 1% current prevalence of bulimia in our study, and no cases of anorexia or other eating disorders were found. Serrano-Blanco et al. reported a 12-month prevalence of bulimia of 0.64% in the primary care population [6]. The data from some other studies had higher prevalence rates, for example, 1-month prevalence of 2.3% in a Madrid primary care study and no cases of anorexia, which was congruent with our study [37]. Another study showed that people with eating disorders sought help using school-based, primary care or specialist services in 40% of cases [47]. These data highlight the importance of screening for eating disorders in primary care settings.

### Factors associated with a current mental disorder

In our study, female sex was significantly associated with any mental disorder, any mood disorder, any anxiety disorder, and suicidality, whereas only any alcohol use disorder was significantly more frequent amongst men. Our findings support earlier findings that the female sex has been identified as an associated factor for psychiatric morbidity, especially depressive and anxiety disorders, both in the primary care and general population [6, 7, 13, 37, 38, 43, 48, 49]. This result indicates that GPs should consider the higher likelihood of common mental disorders amongst women and provide more intense follow-up care for them. However, some studies in the primary health care population in Arab countries and Japan and Israel did not find an association of depression and anxiety disorders with female gender [39, 40, 43]. This fact could be explained by cultural-specific issues; it is possible that in these cultures women are playing a different role in family management.

Those who were not married had a greater risk of any mental disorder, any anxiety disorder and suicidality in our study, which is also consistent with previous studies [43, 50]. The fact that low level of education is an independent risk factor for any mental disorder and any mood disorder was proven in different studies [37, 51]. The data of our study suggested that low education is a risk factor for any mental disorder, suicidality and any anxiety disorder. However, we did not find a significant association between level of education and any mood disorder.

Economically inactive employment status was associated with any mental disorder, any mood disorder, suicidality and any psychotic disorder. A strong association between depressive disorders and economically inactive status has also been found in many other studies [39, 52].

A 6-year longitudinal study of predictors for suicide attempts in patients with major depressive disorder indicated that younger age, lower education, unemployment, insomnia, antidepressant use, a previous suicidal attempt and current suicidal thoughts independently predicted a future suicidal attempt [53].

Another study showed the causal pathways between socioeconomic position and depressive symptoms. The authors indicated that poor socioeconomic conditions lead to depression, which, in turn, can cause further damage to patients’ economic prospects [54].

Living in a small city was associated with any mood disorder and suicidality; in addition, living in the capital of Latvia was a risk factor for any mood disorder and any alcohol use disorder.

However, a recent meta-analysis of data on the prevalence of depression in communities from 30 countries between 1994 and 2014 reports that they did not find a difference in the point prevalence of depression between living in urban versus rural settings [13]. The authors speculated that people in both rural and urban environments have common characteristics that are strongly associated with depression and recommend focusing future research on identifying the risk factors in both particular groups.

Another interesting finding is that living in a small city is a protective factor for any anxiety disorders. A series of studies indicated relationship between urban environment and mental health. Peen et al. exhibit association of living in urban areas (vs. rural) and anxiety disorders [55]. In recent review, social risk factors for mental health in cities were discussed. They include concentrations of low socio-economic status, low social capital, or social segregation. In addition, authors indicated that urban physical environment, such as higher rates of pollution, noise pollution, specific urban designs, or physical threats (accidents, violence) increase the stress levels with negative effects on mental health [56].

Turning to ethnicity-related predictors of each disorder group, we found that being a Russian was a significant protective factor for any anxiety disorder and being a minor ethnicity other than Russian was an associating factor for bulimia (the only eating disorder found in our sample).

The peak association with any mood disorder was being in the 50- to 64-year-old age stratum, and the association with alcohol dependence and abuse decreased significantly with increasing age. This result is congruent with Serrano-Blanco et al., who found that elderly patients presented the lowest rates of any substance used disorders [6]. Ansseau et al. found that the prevalence of major depression increases until the age of 50 years and decreases to lower values after 60 years [7]. The authors suggest that the patients who come to primary care are mostly those with moderate symptoms, whereas those who are severely impaired are visited and treated at home by their GP.

The interpretations presented in different studies of the prevalence rates of mental disorders and associated factors in primary care populations may be related to the measurement instruments used, the structure of the primary care and mental health system, the stigma level of society, the availability of online mental health information, the sociocultural and economic characteristics of the population, the country income and the professional psychiatric knowledge of the family practitioners [12, 30].

GPs should be aware of these predisposing factors rather than screening common mental disorders because of the need to take the sociodemographical factors associated with mental disorders into account.

### Comorbidity

Our results reveal high comorbidity between any mood disorder, suicidality and any anxiety disorder that is consistent with comorbidity rates reported in other studies [2, 6, 23, 37, 41, 57]. The double diagnosis of depressive and anxiety disorders is well known, especially in primary care settings; potentially the different methodology used in various studies may have influenced the prevalence and comorbidity rates [57]. It is important to pay attention to the diagnosis and care of common mental disorders because comorbidities are often associated with greater disability levels in individuals compared to those with a single diagnosis [2, 58].

## Limitations

First, our study demonstrated the prevalence of mental disorders and factors associated with these disorders only in a primary care population, which eliminates the potential to characterize individuals observed in specialized psychiatric outpatient departments and clinical settings. Second, our target population involved persons visiting primary care settings; therefore, they may have been in worse health than the general population. Third, we did not screen patients at home visits. Those patients who were unable to visit a primary centre could have even higher rates of mental disorders. Fourth, another limitation of our study is clinic selection and sample size for some diagnostic categories, such as psychotic disorders and eating disorders. Fifth, we used the MINI as a diagnostic tool for mental disorders; however, a recent publication underlines that a multi-modal assessment approach that involves combined self-report instruments and short-form diagnostic interviews is recommended to screen and identify depressive cases [13]. Finally, due to the cross-sectional design of this study, it is not possible to draw conclusions about the causality of the established links between common mental disorders and associated factors.

## Conclusion

To the best of our knowledge, our study is the first to evaluate the prevalence and associated factors related to mental disorders in the Latvian primary care population. We would like to emphasize that prevalence rates of mental disorders are high. The highest current prevalence of common mental disorders were any mood disorder, suicidality and any anxiety disorder amongst clients of primary care facilities. The considerable rates of comorbidity have been observed.

In terms of clinical implications, the results of our study highlight the importance of screening for depression and anxiety disorders and suicidal risk assessment by GPs in their everyday clinical practice. Strategies for common mental disorder prevention and treatment need to take into consideration their association with sociodemographic disadvantages. Preventive or therapeutic interventions targeting social disadvantages related to health could be beneficial and ultimately reduce healthcare costs [59]. Our findings can support healthcare authorities in developing and implementing successful monitoring programmes and public health policies, promoting mental health and preventing mental disorders in Latvia. There are a number of gaps in our knowledge around primary care and mental health in research that follow from our findings. It would benefit from further research, including evaluation to extend and further test of pharmacotherapy of mental disorders by GPs, exploration of mental and somatic comorbidity, identification the connection between the reason of consultation and existence of mental pathology and evaluation of documented psychiatric pathology in comparison with detected by MINI data.

## Supplementary information

**Additional file 1: Table S1.** Current prevalence of mental disorders and suicidality established by the Mini International Neuropsychiatric Interview.

**Additional file 2: Table S2.** Factors associated with current mental disorders according to sociodemographic variables (adjusted for age and sex).

## Data Availability

The datasets used and analysed during the current study are available from the corresponding author upon reasonable request.

## Abbreviations

CI: Confidence interval
CIDI: Composite International Diagnostic Interview
DSM-IV: Diagnostic and Statistical Manual of Mental Disorders, Fourth Edition
GP: General practitioner
ICD-10: 10th version of the International Classification of Diseases
MINI: Mini-International Neuropsychiatric Interview
OR: Odds ratio
SPSS: Statistical Package for the Social Sciences
WHO: World Health Organization
WMH: World Mental Health
PRIME-MD: The Primary Care Evaluation of Mental Disorders
