# Supplementary material for: Prevalence and associated factors of mental disorders in the nationwide primary care population in Latvia: a cross-sectional study
Source: Ann Gen Psychiatry. 2020 Apr 7;19:25. doi: 10.1186/s12991-020-00276-5 (PMC7137231; doi:10.1186/s12991-020-00276-5)
Supplement: Supplementary file 1 — Additional file 1: Table S1. Current prevalence of mental disorders and suicidality established by the Mini International Neuropsychiatric Interview. [file 12991_2020_276_MOESM1_ESM.docx]

**Additional file 1: Table S1** Current prevalence of mental disorders and suicidality established by the Mini International Neuropsychiatric Interview

|  | **Female/ male ratio** | **Male** | | **Female** | | **Total** | | **P value** |
| --- | --- | --- | --- | --- | --- | --- | --- | --- |
|  |  | **n** | **% (95%CI)** | **n** | **% (95%CI)** | **n** | **% (95%CI)** |  |
| **Any mental disorder** | 3.0 | 133 | 29.7 (25.5-33.9) | 413 | 40.5 (37.5-43.5) | 546 | 37.2 (34.7-39.7) | **<0.001** |
| **Any mood disorder** | 3.9 | 55 | 12.2 (9.2-15.2) | 217 | 21.2 (18.7-23.7) | 272 | 18.4 (16.4-20.4) | **<0.001** |
| Depressive episode | 4.6 | 27 | 6.0 (3.8-8.2) | 124 | 12.0 (10.0-14.0) | 151 | 10.2 (8.7-11.7) | **<0.001** |
| Recurrent depressive disorder | 3.8 | 54 | 11.9 (8.9-14.9) | 205 | 19.9 (17.5-22.3) | 259 | 17.5 (15.6-19.4) | **<0.001** |
| Mania | - | 0 | - | 0 | - | 0 | - | - |
| Hypomania | 1.0 | 1 | 0.2 (0-0.6) | 1 | 0.1 (0-0.3) | 2 | 0.1 (0-0.3) | 0.52 |
| Bipolar disorder I | 1.0 | 1 | 0.2 (0-0.6) | 1 | 0.1 (0-0.3) | 2 | 0.1 (0-0.3) | 0.52 |
| Bipolar disorder II | - | 0 | - | 2 | 0.2 (0-0.5) | 2 | 0.1 (0-0.3) | 1.0 |
| **Suicidality** | 3.5 | 62 | 13.7 (10.5-16.9) | 214 | 20.8 (18.3-23.3) | 276 | 18.6 (16.6-20.6) | **0.001** |
| Low risk | 3.9 | 52 | 11.5 (8.6-14.4) | 202 | 19.6 (17.2-22.0) | 254 | 17.1 (15.2-19.0) | **<0.001** |
| Medium risk | 0.8 | 6 | 1.3 (0.3-2.3) | 5 | 0.5 (0.1-0.9) | 11 | 0.7 (0.3-1.1) | 0.10 |
| High risk | 1.8 | 4 | 0.9 (0-1.8) | 7 | 0.7 (0.2-1.2) | 11 | 0.7 (0.3-1.1) | 0.75 |
| **Any anxiety disorder** | 3.5 | 52 | 11.5 (8.6-14.4) | 183 | 17.7 (15.4-20.0) | 235 | 15.8 (13.9-17.7) | **0.002** |
| Panic disorder | 10.0 | 1 | 0.2 (0-0.6) | 10 | 1.0 (0.4-1.6) | 11 | 0.7 (0.3-1.1) | 0.19 |
| Agoraphobia | 5.0 | 20 | 4.4 (2.5-6.3) | 99 | 9.6 (7.8-11.4) | 119 | 8.0 (6.6-9.4) | **0.001** |
| Social phobia | 3.4 | 16 | 3.5 (1.8-5.2) | 54 | 5.2 (3.9-6.6) | 70 | 4.7 (3.6-5.8) | 0.16 |
| Obsessive-compulsive disorder | 5.7 | 3 | 0.7 (0.1-1.5) | 17 | 1.6 (0.8-2.4) | 20 | 1.3 (0.7-1.9) | 0.13 |
| Posttraumatic stress disorder | 3.3 | 3 | 0.7 (0.1-1.5) | 10 | 1.0 (0.4-1.6) | 13 | 0.9 (0.4-1.4) | 0.77 |
| Generalized anxiety disorder | 3.1 | 22 | 4.8 (2.8-6.8) | 69 | 6.7 (5.2-8.2) | 91 | 6.1 (4.9-7.3) | 0.18 |
| **Any psychotic disorder** | 2.4 | 7 | 1.6 (0.4-2.8) | 17 | 1.7 (0.9-2.5) | 24 | 1.6 (1.0-2.2) | 0.89 |
| **Any alcohol use disorder** | 0.4 | 50 | 11.0 (8.1-13.9) | 20 | 1.9 (1.1-2.7) | 70 | 4.7 (3.6-5.8) | **<0.001** |
| Alcohol dependence | 0.5 | 33 | 7.3 (4.9-9.7) | 16 | 1.6 (0.3-2.4) | 49 | 3.3 (2.4-4.2) | **<0.001** |
| Alcohol abuse | 0.2 | 17 | 3.8 (2.0-5.6) | 4 | 0.4 (0-0.8) | 21 | 1.4 (0.8-2.0) | **<0.001** |
| **Any eating disorder** | 6.5 | 2 | 0.4 (0-1.0) | 13 | 1.3 (0.6-2.0) | 15 | 1.0 (0.5-1.5) | 0.17 |
| Anorexia | - | 0 | - | 0 | - | 0 | - | - |
| Bulimia | 6.5 | 2 | 0.4 (0-1.0) | 13 | 1.3 (0.6-2.0) | 15 | 1.0 (0.5-1.5) | 0.17 |

P value in bold differ significantly from 1.0.
