# Supplementary material for: Prevalence and associated factors of mental disorders in the nationwide primary care population in Latvia: a cross-sectional study
Source: Ann Gen Psychiatry. 2020 Apr 7;19:25. doi: 10.1186/s12991-020-00276-5 (PMC7137231; doi:10.1186/s12991-020-00276-5)
Supplement: Supplementary file 2 — Additional file 2: Table S2. Factors associated with current mental disorders according to sociodemographic variables (adjusted for age and sex). [file 12991_2020_276_MOESM2_ESM.docx]

| **Additional file 2: Table S2** Factors associated with current mental disorders according to sociodemographic variables (adjusted for age and sex) | | | | | | | |
| --- | --- | --- | --- | --- | --- | --- | --- |
|  | OR (95% CI) | | | | | | |
|  | Any mental disorder | Any mood disorder | Suicidality | Any anxiety disorder | Any psychotic disorder | Any alcohol use disorder | Any eating disorder |
| Age |  |  |  |  |  |  |  |
| 18-34 | 1 | 1 | 1 | 1 | - | 1 | 1 |
| 35-49 | 0.90  (0.64-1.27) | 1.04  (0.66-1.64) | 0.83  (0.54-1.25) | 0.86  (0.56-1.33) | 1 | **0.50***  **(0.27-0.90)** | 3.66  (0.45-29.44) |
| 50-64 | 1.17  (0.82-1,67) | **1.64***  **(1.04-2.58)** | 0.89  (0.58-1.38) | 0.99  (0.63-1.55) | 0.30  (0.08-1.05) | **0.24*****  **(0.12-0.52)** | 2.95  (0.34-25.45) |
| 65+ | 1.04  (0.74-1.47) | 1.27  (0.82-1.99) | 0.98  (0.65-1.48) | 0.76  (0.49-1.18) | 0.62  (0.25-1.50) | **0.12*****  **(0.05-0.28)** | 0.45  (0.03-7.27) |
| Sex |  |  |  |  |  |  |  |
| Male | 1 | 1 | 1 | 1 | 1 | 1 | 1 |
| Female | **1.61*** (1.27-2.04)** | **1.92*****  **(1.40-2.65)** | **1.66****  **(1.22-2.25)** | **1.67****  **(1.20-2.32)** | 1.06  (0.43-2.57) | **0.16*****  **(0.09-0.27)** | 2.86  (0.64-12.77) |
| Education |  |  |  |  |  |  |  |
| Higher and unfinished higher education | 1 | 1 | 1 | 1 | 1 | 1 | 1 |
| General or vocational secondary and unfinished secondary | 1.20  (0.94-1.54) | 1.17  (0.74-1.86) | 1.09  (0.80-1.49) | **1.84****  **(1.29-2.63)** | 1.05  (0.39-2.83) | 1.13  (0.63-2.02) | 1.99  (0.55-7.29) |
| 9-year basic, unfinished basic | **1.83** (1.26-2.65)** | 0.99  (0.73-1.34) | **2.38*****  **(1.55-3.67)** | **2.36****  **(1.43-3.90)** | 1.78  (0.46-6.99) | 1.87  (0.80-4.38) | 1.64  (0.16-16.84) |
| Employment status |  |  |  |  |  |  |  |
| Employed | 1 | 1 | 1 | 1 | 1 | 1 | 1 |
| Unemployed | **1.65* (1.04-2.61)** | 1.68  (0.98-2.89) | **1.78***  **(1.03-3.10)** | 1.70  (0.99-2.91) | 1.07  (0.13-8.64) | 1.33  (0.53-3.34) | 0.96  (0.12-7.61) |
| Economically inactive | **1.47* (1.09-2.00)** | **1.51***  **(1.04-2.18)** | **2.39*****  **(1.66-3.45)** | 1.00  (0.67-1.49) | **3.52***  **(1.15-10.79)** | 1.60  (0.79-3.25) | 1.23  (0.33-4.60) |
| Marital status |  |  |  |  |  |  |  |
| Single | **1.59* (1.08-2.36)** | 1.45  (0.87-2.39) | **2.13****  **(1.35-3.34)** | 1.41  (0.87-2.31) | 1.98  (0.43-9.10) | **2.81****  **(1.44-5.49)** | 0.97  (0.11-8.50) |
| Live separately, divorced, widowed | **1.38* (1.08-1.77)** | **1.77*****  **(1.31-2.39)** | **1.50***  **(1.10-2.03)** | 1.22  (0.88-1.69) | 1.39  (0.54-3.58) | **1.98***  **(1.02-3.84)** | 1.25  (0.40-3.91) |
| Married, cohabiting | 1 | 1 | 1 | 1 | 1 | 1 | 1 |
| Place of residence |  |  |  |  |  |  |  |
| Capital (Riga) | 1.23  (0.91-1.67) | **1.89****  **(1.28-2.79)** | 1.42  (0.97-2.09) | 0.84  (0.57-1.24) | 0.37  (0.08-1.75) | **4.06*****  **(1.99-8.28)** | 0.96  (0.17-5.34) |
| Other city | 1.26  (0.99-1.61) | **1.86*****  **(1.34-2.59)** | **1.52****  **(1.11-2.09)** | **0.67***  **(0.50-0.97)** | 1.15  (0.48-2.78) | 1.64  (0.84-3.18) | 1.61 (0.49-5.29) |
| Rural | 1 | 1 | 1 | 1 | 1 | 1 | 1 |
| Ethnicity |  |  |  |  |  |  |  |
| Latvian | 1 | 1 | 1 | 1 | 1 | 1 | 1 |
| Russian | 0.91  (0.72-1.15) | 1.25  (0.93-1.66) | 0.97  (0.72-1.30) | **0.69***  **(0.50-0.96)** | 0.73  (0.28-1.91) | 1.09  (0.62-1.91) | 0.95  (0.28-3.20) |
| Other | 0.88  (0.56-1.38) | 1.13  (0.65-1.95) | 1.11  (0.65-1.90) | 0.77  (0.42-1.41) | 0.58  (0.08-4.43) | 0.77  (0.23-2.64) | **3.93***  **(1.01-15.34)** |

OR: odds ratio in bold differ significantly (* p<0.05, ** p<0.01, *** p<0.001) from 1.0.
